# USP7 promotes non‐small‐cell lung cancer cell glycolysis and survival by stabilizing and activating c‐Abl

**DOI:** 10.1002/ctm2.1509

**Published:** 2023-12-11

**Authors:** Yuanming He, Shuoyi Jiang, Yueya Zhong, Xiaoge Wang, Yaoli Cui, Jingpei Liang, Yuening Sun, Zhigang Zhu, Zhenqian Huang, Xinliang Mao

**Affiliations:** ^1^ Department of Hematology, The Key Laboratory of Advanced Interdisciplinary Studies The First Affiliated Hospital of Guangzhou Medical University Guangzhou P. R. China; ^2^ Guangdong and Guangzhou Key Laboratory of Protein Modification and Degradation, School of Basic Medical Sciences Guangzhou Medical University Guangzhou P. R. China; ^3^ Division of Hematology & Oncology, Department of Geriatrics Guangzhou First People's Hospital, College of Medicine, South China University of Technology Guangzhou Guangdong P. R. China; ^4^ GMU‐GIBH Joint School of Life Sciences, The Guangdong‐Hong Kong‐Macau Joint Laboratory for Cell Fate Regulation and Diseases Guangzhou Medical University Guangzhou P. R. China

**Keywords:** c‐Abl, hexokinase‐2, non‐small‐cell lung cancer, the ubiquitin‐proteasomal pathway, USP7

## Abstract

**Background:**

Abelson tyrosine kinase (c‐Abl) is frequently mutated and highly expressed, and promotes non‐small‐cell lung cancer (NSCLC) survival, metastasis and tumorigenesis. c‐Abl could also be modified through ubiquitination, but the underlying mechanism is not well understood.

**Methods:**

Mass spectrometry assays were performed to search c‐Abl deubiquitination enzymes. The molecular mechanism was determined using Co‐IP assays, pull‐down assays, Western blotting upon gene knockdown or overexpression. Cell lines and animal models were used to investigate the role of c‐Abl and USP7 in NSCLC. EdU staining assay and Transwell assay were performed to evaluate the proliferation and migration ability of NSCLC cells, respectively.

**Results:**

Ubiquitin‐specific protease 7 (USP7) is found to upregulate c‐Abl via the deubiquitinase screen. USP7 interacts with c‐Abl and decreases its K48‐linked polyubiquitination, thereby increasing the stability of c‐Abl. In addition to the wild‐type one, c‐Abl mutants can also be deubiquitinated and stabilized by USP7. Moreover, USP7 promotes c‐Abl accumulation in cytoplasm by increasing its binding to 14‐3‐3α/β and activates the oncogenic c‐Abl signalling pathway. Furthermore, the USP7/c‐Abl axis promotes NSCLC cell glycolysis by direct phosphorylating and stabilizing hexokinase‐2 (HK2). Knockdown of USP7 or c‐Abl suppresses NSCLC cell glycolysis and reduces lactate production. Further studies revealed that overexpression of USP7 facilitates NSCLC cell growth and metastasis as well as xenograft growth in nude mice, while these activities are suppressed with USP7 or c‐Abl being knocked down.

**Conclusions:**

USP7 is a deubiquitinase of c‐Abl and upregulates its oncogenic activity. USP7 promotes NSCLC cell metabolism by activating c‐Abl and HK2. Targeting the USP7/c‐Abl/HK2 axis might be a potential strategy to the precision therapy of NSCLC.

## Graphical abstract

1. USP7 is a putative deubiquitinase of c‐Abl and increases its stability.

2. USP7 increases the binding of c‐Abl and 14‐3‐3α/β, therefore arresting c‐Abl in cytosol.

3. c‐Abl phosphorylates and stabilizes HK2 protein, thereby facilitating cell glycolysis and promotes NSCLC survival and growth.

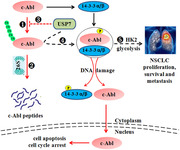

## 1 INTRODUCTION

Lung cancer is a malignant tumour originating from the alveolar epithelium, and it has become the leading cause of cancer‐related deaths worldwide, especially in non‐small‐cell lung cancer (NSCLC).^1^, ^2^ In the past decades, significant progress has been made in targeted therapy for patients with NSCLC,^2^, ^3^ the most well‐known of which is mutated epidermal growth factor receptor (EGFR). EGFR mutations are found in nearly 50% of Asian patients with advanced NSCLC, and these mutants display stronger kinase activity than the wild‐type ones.^4^ EGFR‐targeting drugs such as gefitinib and osimertinib have been successfully used in clinic.^5^ However, the 5‐year survival rate of patients with NSCLC remains low, and it is urgent to extend our understanding of the molecular pathophysiology of NSCLC and to discover novel therapeutic targets.

Abelson tyrosine kinase (c‐Abl) is a member of non‐receptor tyrosine kinases and is widely distributed in human tissues and cells, and its kinase activity is strictly regulated. BCR‐ABL, the fusion kinase of B‐cell receptor (BCR) and c‐Abl resulting from the Philadelphia chromosome, is a driver gene in initiating chronic myeloid leukaemia (CML) and promoting the malignant progression of CML.^6^ Unlike haematologic diseases, c‐Abl is not found to form a fusion gene in solid tumours, but it regulates the progression of solid tumours through abnormal expression or over‐activation.^7^ In addition, c‐Abl mutants, such as T315I and G340L, are more active in tumour progression and drug resistance.^7^ Moreover, c‐Abl activates various substrates and promotes the epithelial–mesenchymal transition (EMT) involved in migration and invasion and the metastatic cascade in various solid cancers including NSCLC,^8^ but the detailed mechanism of c‐Abl remains largely unknown.

It is known that the c‐Abl protein can be modified by ubiquitination and degraded via proteasomes under direction of a specific ubiquitin ligase.^7^, ^9^, ^10^ Meanwhile, this process is reversible and the conjugated ubiquitin chain(s) could be hydrolyzed by a certain deubiquitinase (Dub).^11^ However, there are no proven Dubs reported for c‐Abl ubiquitination and stability. USP7, also known as herpesvirus‐associated ubiquitin‐specific protease (HAUSP) or ubiquitin carboxyl‐terminal hydrolase 7, is one of the most studied deubiquitinases, and a myriad of substrate proteins have been assigned to USP7, including PTEN, P53, RNF6,^12^ and BCR‐ABL, c‐Myc and many others.^13^ In the present study, we found USP7 as a deubiquitinase that can prevent c‐Abl from K48‐linked polyubiquitination and proteasomal degradation. The USP7/c‐Abl axis promotes NSCLC cell glycolysis process by stabilizing hexokinase‐2 (HK2) protein, therefore promoting NSCLC tumour progression.

## 2 MATERIALS AND METHODS

### 2.1 Cell culture

A549, H1299 and H460 were purchased from Procell Life Science & Technology Co., Ltd. Cells were cultured in RPMI‐1640 medium, as described previously.^14^

### 2.2 Plasmids and antibodies

USP7 and its truncates were constructed as described previously.^6^ HA‐c‐Abl was kindly provided by Dr. Jinbo Cheng (Chinese Academy of Sciences, Beijing, China). The truncates and mutants of c‐Abl were generated by direct mutagenesis or PCR with specific primers flapped with a Myc tag. Monoclonal anti‐HA (Cat. #561‐5), anti‐Myc (Cat. #M192‐3) and anti‐Flag (Cat. #PM020‐7) were obtained from MBL. Antibodies against GAPDH (Cat. #10494‐1‐AP), HK2 (Cat. #66974‐1‐Ig), GLUT2 (Cat. #20436‐1‐AP), PKM2 (Cat. #15822‐1‐AP) and Lamin B1 (Cat. #12987‐1‐AP) were purchased from Proteintech Group, Inc. Antibodies against AKT (Cat. #S4691), p‐AKT(T473) (Cat. #S4060), STAT3 (Cat. #S9139), p‐STAT3(Y705) (Cat. #S9145), STAT5 (Cat. #S25656), p‐STAT5(Y694) (Cat. #S4322), CRKL (Cat. #S38710), p‐CRKL(Y207) (Cat. #S3181), Lyn (Cat. #S2796), p‐Lyn(Y507) (Cat. #S2731), FOXM1 (Cat. #S20459), PARP (Cat. #S9532), LDHA (Cat. #S3582), HSP90 (Cat. #S4877), c‐Myc (Cat. #S18583), p‐c‐Abl(T735) (Cat. #S2864) and USP7 (Cat. #S4833) were purchased from Cell Signalling Technology, Inc. Antibodies against c‐Abl (Cat. #sc‐56887), p‐c‐Abl(Y412) (Cat. #sc‐101626) and 14‐3‐3α/β (Cat. #sc‐25276) were obtained from Santa Cruz Biotechnology.

### 2.3 Chemicals

Cycloheximide (CHX, Cat. #66‐81‐9) was purchased from Sigma‐Aldrich Chemicals P5091 (Cat. #S7132) and doxorubicin (DOX, Cat. #E2516) was purchased from Selleck Chemicals Inc. Cisplatin (Cat. #15663‐27‐1) was purchased from Solarbio.

### 2.4 Small interfering RNAs and short hairpin RNAs

The small interfering RNAs (siRNAs) and short hairpin RNAs (shRNAs) were synthesized by Ribobio.^6^ The two effective target sequences of siRNAs for USP7 were: 5′‐GAGCGACCTTACCCAAGTT‐3′ and 5′‐TAAGGACCCTGCAAATTAT‐3′. siRNA sequences for c‐Abl were 5′‐TGATCAAGAAGAAGAAGAA‐3′ and 5′‐TCAACAAACTGGAGAATAA‐3′. Specific target sequences of shRNAs for USP7 were 5′‐GTCCTATATCCAGTGTAAA‐3′ and 5′‐TATCTATTGACTGCCCTTT‐3′. The control siRNA and shRNA sequences were 5′‐UUCUCCGAACGUGUCACGUdT‐3′ and 5′‐TTCTCCGAACGTGTCACGT‐3′, respectively.

### 2.5 CRISPR genome editing

To generate c‐Abl or USP7‐knockout NSCLC cells, the specific sgRNA sequences were obtained by using the CHOPCHOP (http://chopchop.cbu.uib.no/).^15^ The single‐guide RNAs (sgRNAs) of c‐Abl were 5′‐CAAGTGGGAGATGGAACGCA‐3′ and 5′‐CTCGTCCTCCAGCTGTTATC‐3′; sgRNAs of USP7 were 5′‐AGTGATGGACACAACACCG‐3′ and 5′‐CTCGTCCTCCAGCTGTTATC‐3′. The control sgRNA was 5′‐CTCTGCTGCGGAAGGATTCG‐3′.

### 2.6 Affinity purification‐coupled tandem mass spectrometry (AP/MS/MS)

HEK293T cells expressing a HA‐c‐Abl plasmid were subjected to protein purification as described previously^12^ and the extracted proteins were then incubated with HA‐tagged beads for 16 h at 4°C for immunoprecipitation (IP) assay. The prepared protein samples were Coomassie blue staining (Beyotime) before the bands were prepared for MS/MS assay.^12^, ^16^

### 2.7 Immunoblotting

Cell lysates subjected to immunoblotting (IB) assay were described previously.^12^

### 2.8 Immunoprecipitation

Upon treatment, cell lysates were cleared by centrifugation before IP assay, as described previously.^16^

### 2.9 Immunoflourescence assay

Cells expressing USP7 or control plasmid were plated into 24‐well plate for immunoflourescence (IF) assay, as described previously by using a confocal microscopy.^16^

### 2.10 EdU incorporation assay

Cell proliferation activity was measured by staining with the Cell‐Light EdU Apollo567 In Vitro Kit (Ribobio), as described previously.^14^

### 2.11 Isolation of the nuclear and the cytoplasmic fractions

After cells were washed and harvested in cold PBS, isolation of the cytoplasmic and nuclear fractions was performed by using a Nuclear and Cytoplasmic Protein Extraction Kit (Beyotime, Cat. #P0027) according to the manufacturer's instruction.

### 2.12 Cycloheximide chase assay

Upon transfection with specific plasmids, A549 and H1299 cells were then treated with CHX (100 μg/mL) for a certain period before lysis. Extracted proteins were then prepared for IB assays, as described previously.^14^

### 2.13 Reverse‐transcription polymerase chain reaction

RNA extraction and the reverse‐transcription polymerase chain reaction (RT‐PCR) procedure were carried out, as reported previously.^14^ The specific primers for USP7 were: 5′‐TTTTGTGCGAAATCTGCC‐3′ (forward) and 5′‐AATCCCACGCAACTCCAT‐3′ (reverse); for c‐Abl, 5′‐ACATCACGCCAGTCAACAG‐3′ (forward) and 5′‐CTCGGAGGAGACGTAGAGC‐3′ (reverse). The primers for GAPDH control were described previously.^6^

### 2.14 Lactate and pyruvate assays

The concentrations of lactate and pyruvate were detected by using a specific kit from Beijing Boxbio Science & Technology Co., Ltd., as instructed by the manufacturer.

### 2.15 Immunohistochemistry staining

Immunostaining was conducted using a DAB kit (Servicebio) after the tissues were embedded by paraffin waxes and mounted onto slides. The expression of c‐Abl and USP7 in NSCLC patient's tissues (*n* = 22) were analyzed by immunohistochemistry (IHC) assay, as described previously.^17^ The characteristics of NSCLC patients are shown in Table 1. To compare the expression levels of the proteins in each patient, the intensity of staining was scored according to a semiquantitative four‐grade scale, as described previously.^18^ The study on primary NSCLCs was approved by the Ethical Committee of Guangzhou Medical University.

**TABLE 1:** Characteristics of NSCLC patients.

No.	Age	Gender	Type	Grade	TNM	Stage	Source	Lymphatic metastasis
1	62	Male	Squamous carcinoma	3	T3N2M0	IIIB	Primary	Yes
2	73	Male	Squamous carcinoma	3	T2bN0M0	IIA	Primary	No
3	70	Male	Squamous carcinoma	3	T3N1M0	IIIA	Primary	Yes
4	52	Male	Squamous carcinoma	3	T1cN1N0	IIB	Primary	Yes
5	58	Male	Squamous carcinoma	3	T3N0M0	IIB	Primary	No
6	69	Female	Squamous carcinoma	3	T3N2M0	IIIB	Primary	Yes
7	70	Male	Squamous carcinoma	3	T3N1M0	IIIA	Primary	Yes
8	68	Male	Adenocarcinoma	2	T2N1M0	IIB	Primary	Yes
9	66	Male	Adenocarcinoma	2	T2N1M0	IIB	Primary	Yes
10	63	Male	Adenocarcinoma	3	T3N2M0	IIIA	Primary	Yes
11	56	Male	Adenocarcinoma	2	T2aN2M0	IIIA	Primary	Yes
12	59	Female	Squamous carcinoma	2	T4N2M0	IIIB	Primary	Yes
13	56	Male	Squamous carcinoma	2	T3N2M0	IIIB	Primary	Yes
14	69	Female	Squamous carcinoma	2	T3N2M0	IIIB	Primary	Yes
15	61	Male	Squamous carcinoma	2	T2bN0M0	IIA	Primary	No
16	68	Male	Squamous carcinoma	2	T4N0M0	IIIA	Primary	No
17	66	Male	Squamous carcinoma	2	T3N0M0	IIB	Primary	No
18	52	Male	Squamous carcinoma	2	T1bN0M0	IA2	Primary	No
19	63	Male	Squamous carcinoma	2‐3	T3N1M0	IIIA	Primary	Yes
20	56	Female	Adenocarcinoma	2	T1bN0M0	IA	Primary	No
21	60	Female	Adenocarcinoma	2	T1N0M0	IA	Primary	No
22	52	Male	Adenocarcinoma	2	T2N0M0	IB	Primary	No

Abbreviation: TNM: tumour, regional lymph nodes and distant metastasis.

### 2.16 HK2 activity assay

HK2 activity was measured by using a specific test kit obtained from Solarbio, as suggested. The activity of HK2 in control group was normalized to 100%.

### 2.17 Xenografted tumour models in vivo

The NSCLC xenografts with A549 cells stably expressing USP7 or H1299 cells infected with sgUSP7 and their counterparts were established at a density of 2 × 10^6^ cells per injection into female nude Balb/c mice from Beijing Vital River Laboratory Animal Technology Co., Ltd. Mice were assigned randomly into control and treatment groups when tumours were palpable. After completing the experiments, mice were sacrificed in CO_2_, and tumour tissues were dissected and prepared for further studies as described previously.^16^ This animal experiment was approved by the Review Board for Animal Welfare and Ethics of Guangzhou Medical University (#2019‐142).

### 2.18 Statistics

Statistical difference between the control and the experimental groups was analyzed by Student's *t*‐test. The Pearson correlation coefficient was analyzed for the correlation between c‐Abl and USP7 in the patient samples. All experiments were run in triplicates, except for specific indication. *p*‐Values less than .05 were considered statistically significant.

## 3 RESULTS

### 3.1 c‐Abl is overexpressed in NSCLC tissues and confers resistance to anti‐NSCLC drugs

**FIGURE 1:**
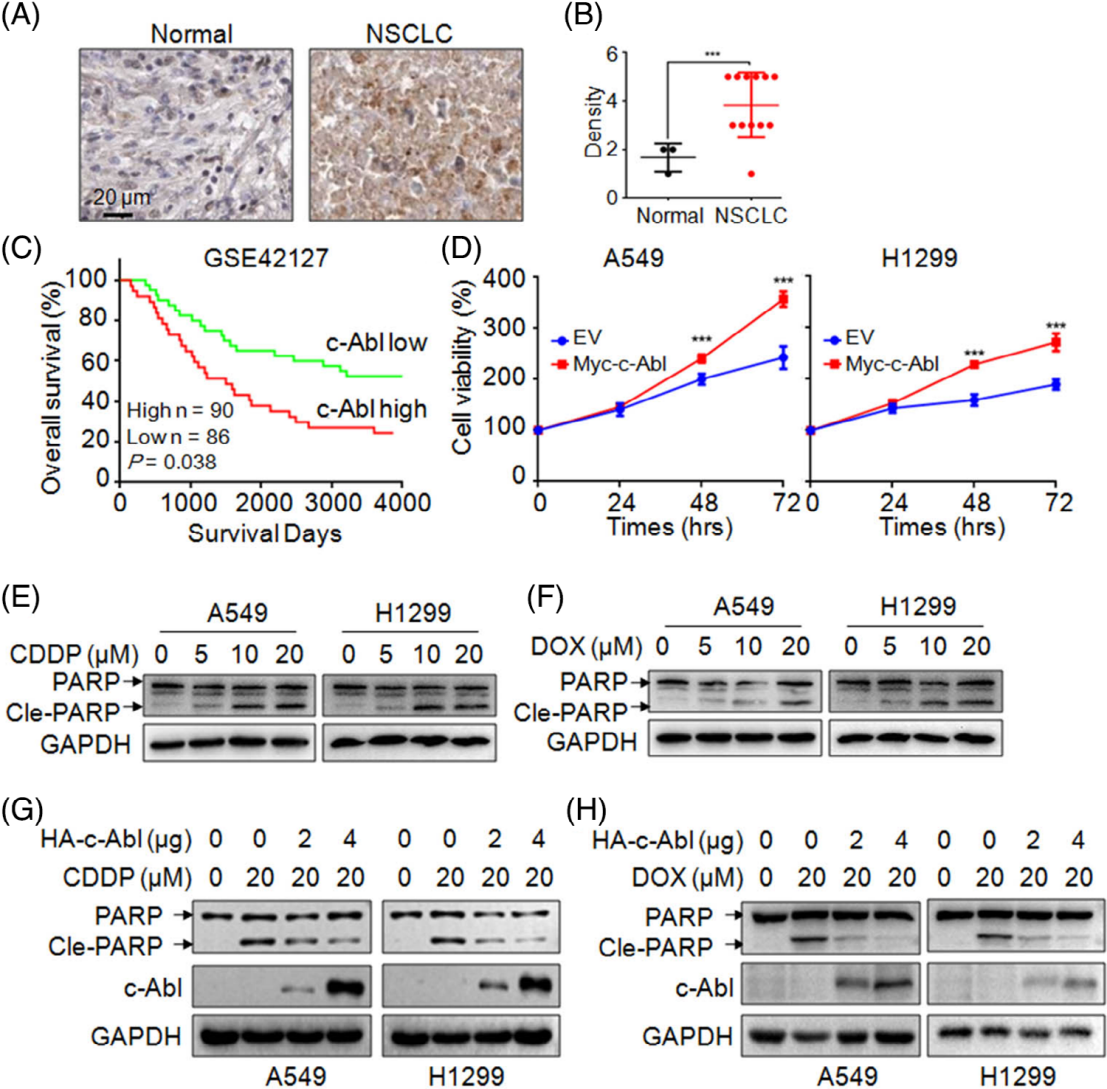
c‐Abl is overexpressed in NSCLC tissues and increases NSCLC cell proliferation and contributes to resistance to anti‐NSCLC drugs. (A and B) IHC analysis of the expression level of c‐Abl in normal lung tissues and tumours cited from the Human Protein Atlas. Representative images are shown. ***p < .001, Student's *t*‐test. (C) Data from PROGgeneV2 showing the survival rate of NSCLC patients classified by c‐Abl expression. (D) NSCLC cells A549 and H1299 were transfected with Myc‐c‐Abl plasmids for 24 h, followed by MTT assay. (E and F) A549 and H1299 were treated with CDDP (E) and DOX (F) for 24 h, followed by IB assay. (G and H) NSCLC cells were transfected with increased c‐Abl for 24 h, followed by CDDP (G) or DOX (H) treatment for 24 h, and cells were then collected for IB assay.

To find out the expression level of c‐Abl in NSCLC tissues, we analyzed the Human Protein Atlas (https://www.proteinatlas.org/), from which c‐Abl was found highly expressed in NSCLC. The representative image of IHC assays and the score was shown in Figure 1A and B, respectively. The expression of c‐Abl was higher in tumours than that in normal lung tissues. Subsequently, we analyzed the relationship of the survival rate and c‐Abl expression based on the TCGA data from PROGgeneV2 (http://www.progtools.net/gene/index.php) and found that high expression of c‐Abl was significantly associated with low overall survival rate (poor prognosis, Figure 1C). We further demonstrated that c‐Abl promoted NSCLC cell proliferation (Figure 1D) and conferred resistance to anti‐NSCLC drugs, such as CDDP or DOX. As shown in Figure 1E–H, CDDP and DOX induced NSCLC cell apoptosis, but the apoptotic levels were markedly reduced by overexpression of c‐Abl. These results demonstrated that c‐Abl plays an oncogenic role to facilitate NSCLC cell growth and chemoresistance.

### 3.2 USP7 stabilizes c‐Abl in NSCLC cells

**FIGURE 2:**
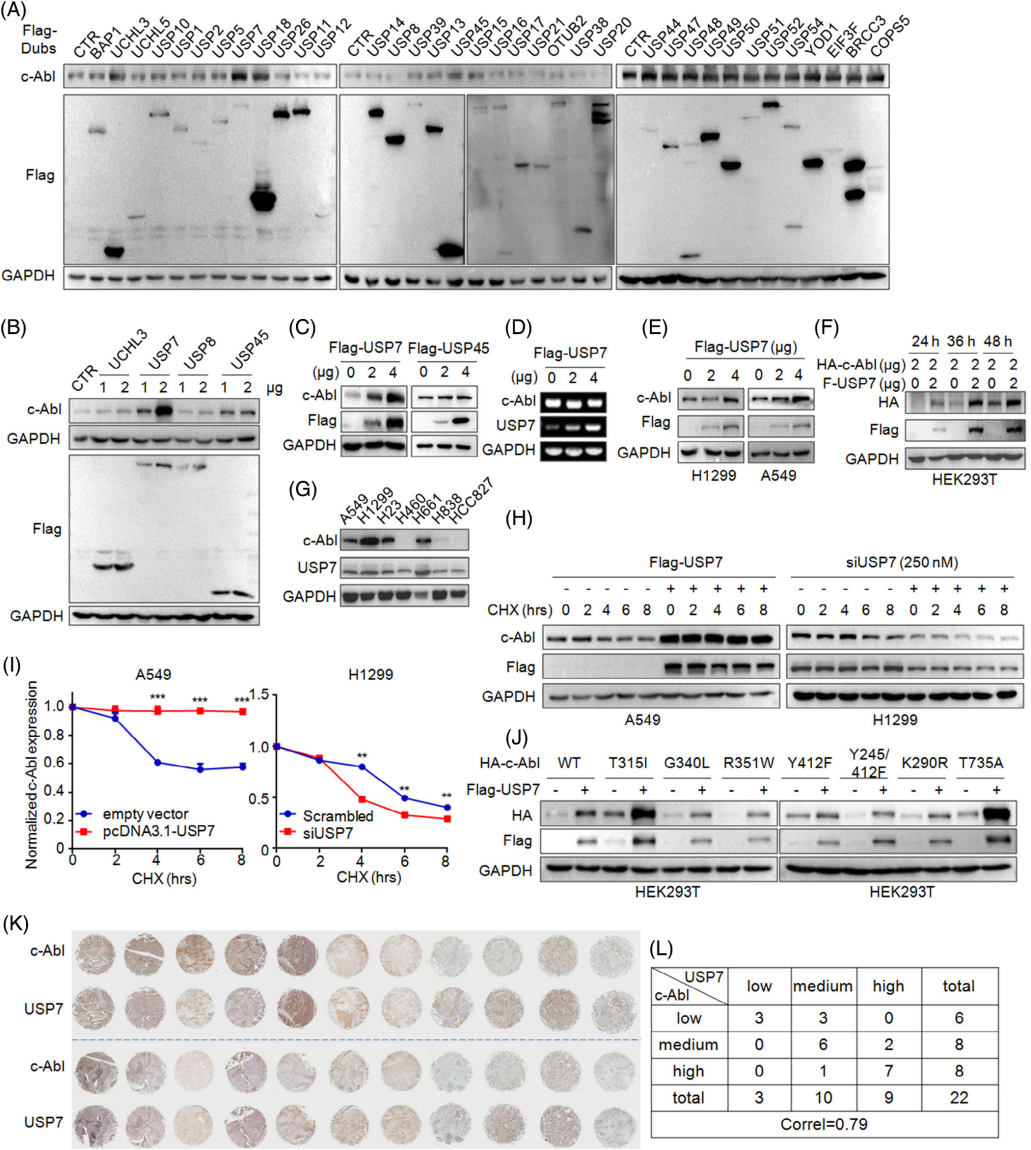
USP7 stabilizes c‐Abl protein but not its mRNA. (A) HEK293T cells were transfected with different deubiquitinases for 48 h, followed by IB assay as indicated. (B) HEK293T cells were transfected with four candidate Dubs for 48 h, followed by IB as indicated. (C) After being transfected with USP7 or USP45 plasmids for 48 h, HEK293T cells were applied for IB assays to measure c‐Abl. (D) The c‐Abl and USP7 RNAs were measured by using RT‐PCR. (E) NSCLC cells were transfected with Flag‐USP7 plasmids for 48 h, followed by IB assay. (F) HEK293T cells were transfected with USP7 and c‐Abl plasmids for indicated periods, followed IB assay. (G) The c‐Abl and USP7 expression profiles were evaluated by IB in NSCLC cell lines. (H) A549 cells were transfected with USP7 plasmids, and H1299 cells were transfected with USP7 siRNA for 24 h, followed by cycloheximide (CHX) treatment before being subjected to IB assays. (I) c‐Abl stability was analyzed by densitometry based on H. (J) HEK293T cells were co‐transfected with Flag‐USP7, c‐Abl or its mutant plasmids for 48 h, followed by IB assays. (K) A NSCLC tissue array was subjected to IHC assays as indicated in the Methods section. (L) The score analysis on the IHC results in I. The correlation between the expression of c‐Abl and USP7 was analyzed by *Pearson's correlation coefficient*.

c‐Abl is overexpressed in NSCLC, and it has been reported that c‐Abl can be degraded through the ubiquitin–proteasome pathway (UPP) in the direction of ubiquitin ligase SMURF1,^10^ but its Dub remains elusive. To identify the potential Dub(s) of c‐Abl, a panel of 36 deubiquitinating enzymes was co‐transfected with c‐Abl, followed by measurement of c‐Abl protein. It revealed that UCHL3, USP7, USP8 and USP45 increased c‐Abl protein level (Figure 2A). The subsequent confirmation assay showed USP7, but not the other Dubs markedly increasing c‐Abl protein (Figure 2B,C). Moreover, USP7 did not alter c‐Abl mRNA (Figure 2D). USP7 increased c‐Abl protein in a time‐ and concentration‐dependent manner (Figure 2E,F). Moreover, the expression profiling assay on c‐Abl and USP7 in NSCLC cell lines revealed that c‐Abl expression was closely related to USP7 (Figure 2G). Furthermore, USP7 significantly increased the half‐life of c‐Abl in NSCLC cells, when USP7 was depleted, c‐Abl protein was quickly decreased (Figure 2H,I). Given c‐Abl is frequently mutated in NSCLC,^7^ we wondered whether USP7 affected the stability of c‐Abl mutants. The IB assay showed that USP7 upregulated the protein levels of all c‐Abl mutants (Figure 2J). Lastly, we analyzed the expression levels of both USP7 and c‐Abl in a tissue array derived from NSCLC patients (Table 1). The IHC analysis and scoring assay showed that in each individual patient, the c‐Abl protein level was positively associated with the USP7 expression level (Figure 2K,L) as assayed by the Pearson's coefficient of correlation. Therefore, USP7 regulates the stability of c‐Abl independent of its mutation status.

### 3.3 c‐Abl physically interacts with USP7

**FIGURE 3:**
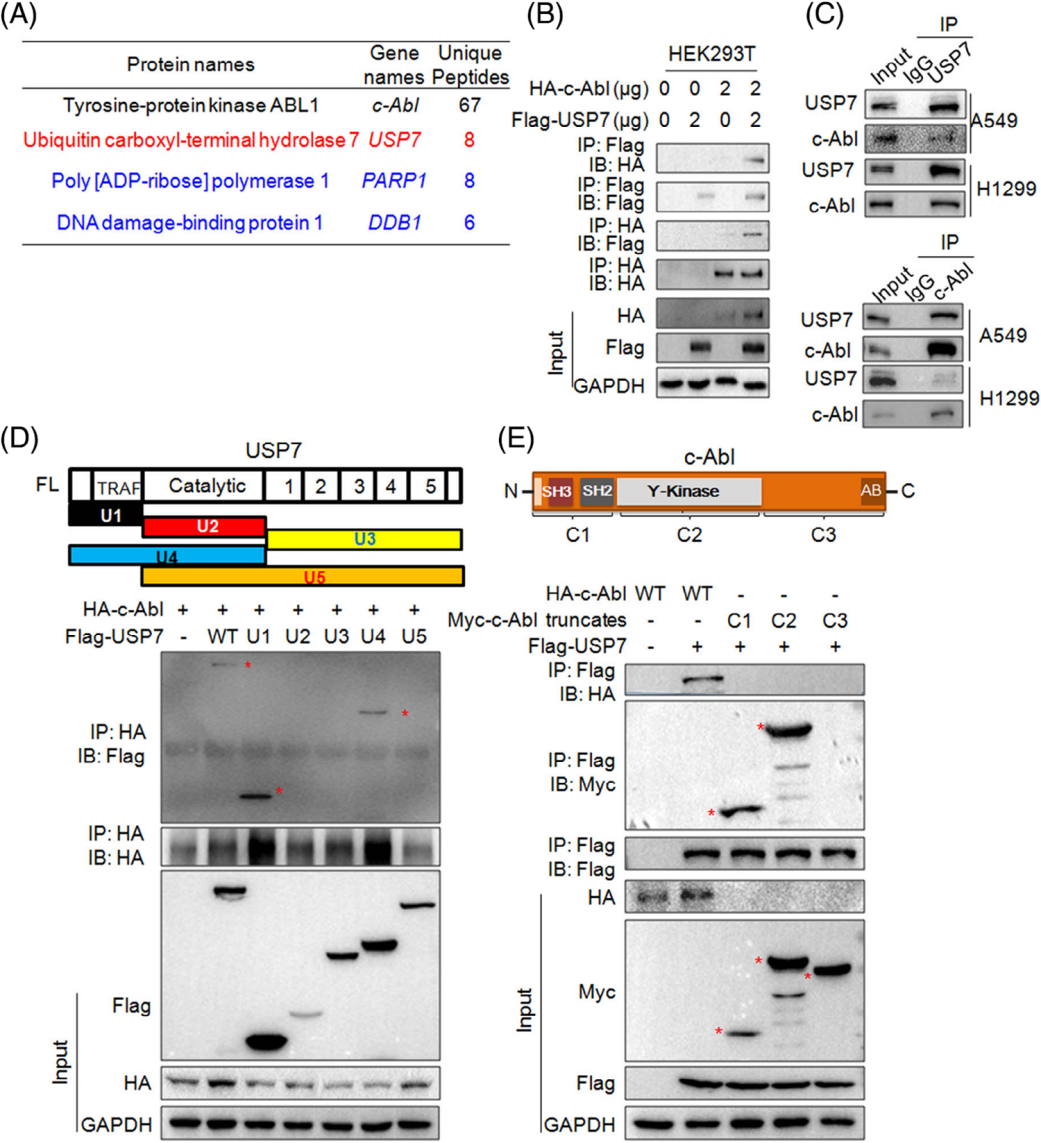
USP7 interacts with c‐Abl via the TRAF domain. (A) HEK293T cells were transfected with a HA‐c‐Abl plasmid for 48 h, followed by immunoprecipitation (IP) with an anti‐HA antibody to enrich and purify c‐Abl‐interacting complexes. The eluted proteins were then subjected to trypsin digestion and tandem mass spectrometric (MS/MS) analysis. (B) HEK293T cells were co‐transfected with Flag‐USP7 and HA‐c‐Abl plasmids for 48 h, cell lysates were then subjected to IP/IB assays as indicated. (C) Cell lysates from NSCLC cell lines A549 and H1299 were incubated with an anti‐USP7 or c‐Abl antibody overnight, followed by IB with an anti‐c‐Abl or anti‐USP7 antibody as indicated. (D) HEK293T cells were co‐transfected with HA‐c‐Abl, Flag‐USP7 or its truncate plasmids for 48 h before being prepared for IP/IB assays as indicated. (E) HEK293T cells were co‐transfected with Flag‐USP7, HA‐c‐Abl or its truncate plasmids for 48 h before being prepared for IP/IB assays. *Specific bands.

To further find out the association of USP7 and c‐Abl, we performed an AP/MS as described previously,^19^ and found USP7, PARP1 and DDB1 in the c‐Abl interactome (Figure 3A), of which the latter two (DDB1 and PARP1) have been reported to interact with c‐Abl.^20^ To confirm the interaction between USP7 and c‐Abl, we co‐transfected USP7 and c‐Abl plasmids into HEK293T cells. The subsequent IP/IB assays showed that c‐Abl was co‐immunoprecipitated with USP7 (Figure 3B). Moreover, the reciprocal IP/IB assays further demonstrated that USP7 interacted with c‐Abl in both A549 and H1299 cells (Figure 3C). Furthermore, the co‐transfection and IP/IB assays on the truncates of USP7 and c‐Abl showed that USP7 interacted with c‐Abl via its N‐terminal TRAF domain (Figure 3D), while c‐Abl interacted with USP7 via its N‐terminal SH3/SH2 and Y‐kinase domains (Figure 3E). The above results collectively demonstrated that USP7 interacts with and stabilizes c‐Abl.

### 3.4 USP7 stabilizes c‐Abl through its deubiquitination activity

**FIGURE 4:**
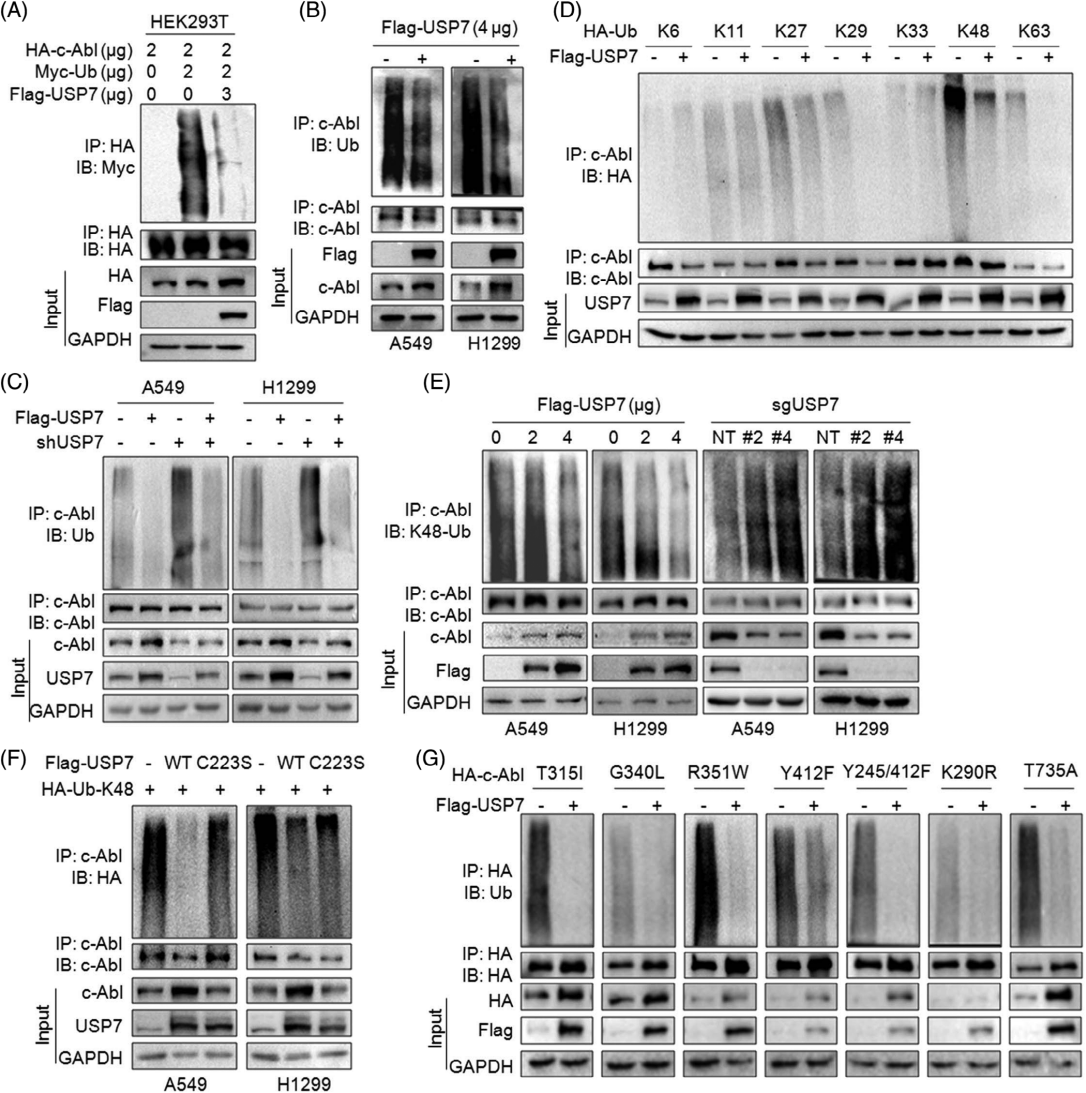
USP7 prevents c‐Abl from K48‐linked polyubiquitination. (A) HEK293T cells were co‐transfected with Flag‐USP7, Myc‐Ub and HA‐c‐Abl plasmids for 48 h, cell lysates were then subjected to IP/IB assays as indicated. (B) NSCLC cells were transfected with a Flag‐USP7 plasmid for 48 h, followed by IP/IB assays to measure the ubiquitination level of c‐Abl. (C) A549 and H1299 cells were co‐transfected with USP7 and its shRNA for 48 h, followed by IP/IB assays as indicated. (D) HEK293T cells were co‐transfected with Flag‐USP7 and each Ub mutant for 48 h, followed by IP/IB assays. (E) NSCLC cells were transfected with a USP7 plasmid or knocked out of USP7 for 48 h, followed by IP/IB assays to measure the K48‐linked ubiquitination level of c‐Abl. (F) NSCLC cells were co‐transfected with K48‐Ub, USP7 or the USP7‐C223S plasmid for 48 h, followed by IP/IB assays as indicated. (G) HEK293T cells were co‐transfected with plasmids of Flag‐USP7 and c‐Abl mutants for 48 h before being subjected to IP/IB assays as indicated.

Given USP7 is a Dub, we wondered whether USP7 stabilizes c‐Abl by preventing its polyubiquitination. To this end, we performed a series of ubiquitination assays on c‐Abl, and found that c‐Abl ubiquitination levels were significantly reduced in the presence of USP7 (Figure 4A,B). Conversely, when the specific shRNA of USP7 was transfected into cells, c‐Abl was presented with heavy ubiquitination (Figure 4C). On the other hand, after co‐expressing ubiquitin plasmids with specific mutations, the subsequent IP/IB assays showed that USP7 specifically prevented c‐Abl from K48‐linked polyubiquitination (Figure 4D), and this result was further confirmed in NSCLC cells by overexpressing or knocking out USP7 (Figure 4E). Notably, when Cys223, an essential amino acid residue for USP7 deubiquitinase activity, was mutated,^6^ USP7 lost its deubiquitinating activity against c‐Abl ubiquitination in both NSCLC cell lines (Figure 4F). Furthermore, c‐Abl is reported with multiple mutations in NSCLC patients and these mutants display more potent oncogenic activity,^7^ we thus next evaluated the ubiquitination on c‐Abl mutants by USP7. The results showed that USP7 stabilized c‐Abl mutants along with decreased polyubiquitination (Figure 4G). Collectively, these results concluded that USP7 promotes c‐Abl stability by deubiquitinating c‐Abl independent of its mutation status.

### 3.5 USP7 promotes c‐Abl retention in cytosol by promoting the interaction between c‐Abl and 14‐3‐3α/β

**FIGURE 5:**
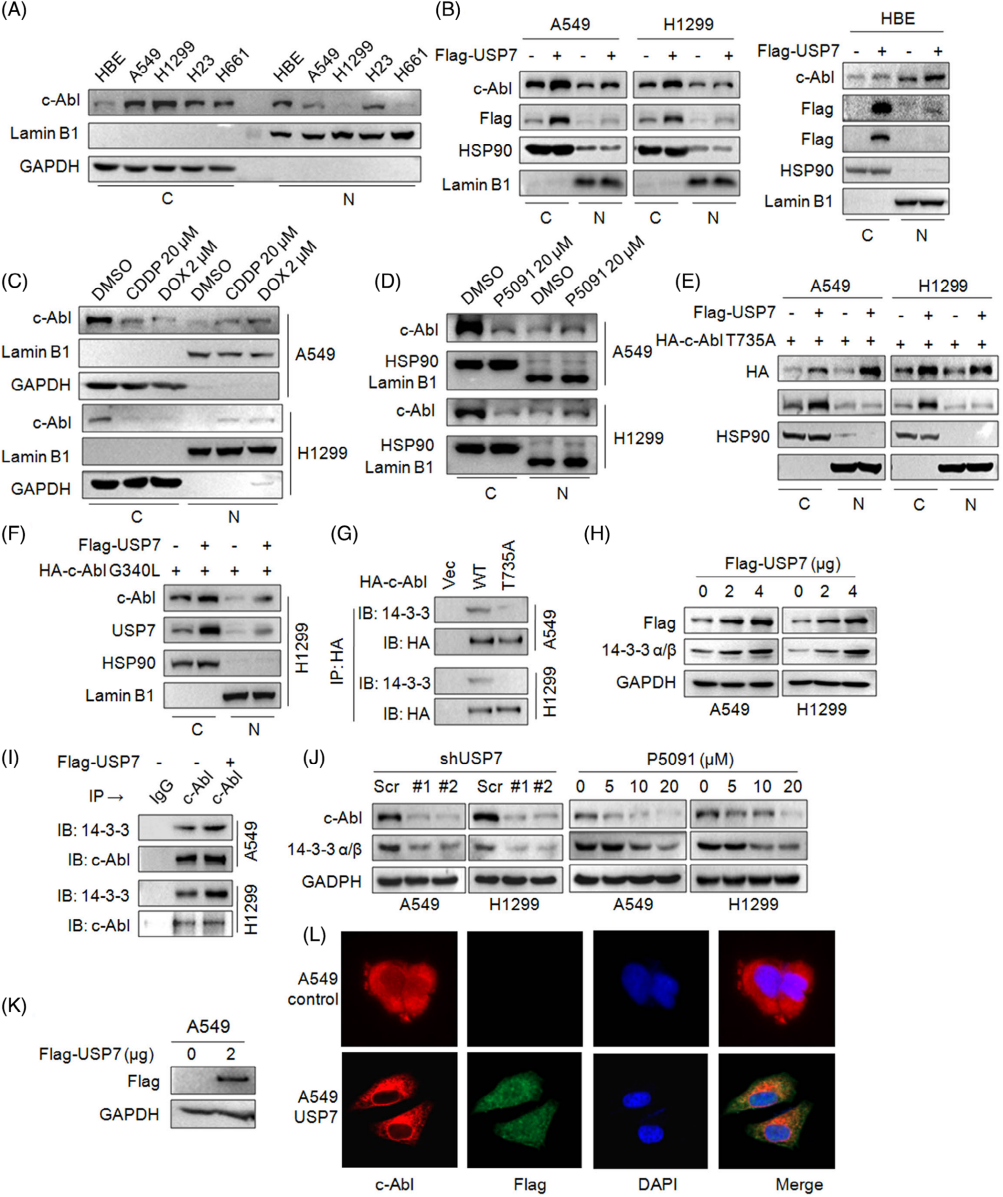
USP7 upregulates 14‐3‐3α/β expression and promotes their interaction with c‐Abl, thereby retaining c‐Abl in cytosol. (A) The cytosol and nuclear distribution of c‐Abl in healthy bronchial epithelial cells (HBE) and NSCLC cells was measured by IB. (B) A549, H1299 and HBE were transfected with a USP7 plasmid for 48 h. The cytosol and nuclear fractions were isolated for IB assays as indicated. (C and D) A549 and H1299 cells were treated with CDDP or DOX (C) or P5091 (D) for 24 h. The cytosol and nuclear fractions were isolated for IB assays as indicated. (E and F) A549 and H1299 were co‐transfected with a USP7 plasmid and a c‐Abl‐T735A (E) or a c‐Abl‐G340L (F) plasmid for 48 h. The cytosol and nuclear fractions were isolated for IB assays as indicated. (G) NSCLC cells were transfected with c‐Abl or c‐Abl‐T735A plasmids for 48 h, followed by IP/IB assays as indicated. (H) NSCLC cells were transfected with a USP7 or empty vector for 48 h, followed by IP/IB assays as indicated. (I) NSCLC cells were transfected with a Flag‐USP7 plasmid for 48 h, followed by IB assay as indicated. (J) A549 and H1299 cells were infected with shUSP7 lentivirus or treated with P5091 for 48 h, followed by IB assay as indicated. (K and L) A549 cells were transfected with a USP7 plasmid for 48 h (K), followed by immunofluorescence assay and confocal microscopy analysis (L).

The c‐Abl protein contains three nuclear localization signals (NLS) and one nuclear export signal (NES), it can shuttle between cytosol and nuclei upon cell stress and DNA damage.^21^ The nuclear c‐Abl is activated by DNA damage repair reaction and then induces cell cycle arrest and cell apoptosis.^22^ In contrast, the cytoplasmic c‐Abl is activated by various oncogenic tyrosine kinases, therefore it promotes cancer cell proliferation, migration and adhesion.^23^, ^24^ Our above study showed that USP7 interacts with c‐Abl, and we wondered whether this interaction also affects c‐Abl cellular distribution. To this end, we first analyzed the distribution of c‐Abl in NSCLC cell lines and healthy lung epithelial cells. It showed that more c‐Abl was found in cytosol of NSCLC cells, in contrast, more c‐Abl in nuclei of HBE, a healthy human bronchial epithelium (Figure 5A). However, when USP7 was transfected, c‐Abl was markedly increased in cytosol in NSCLC but not in HBE cells (Figure 5B). Interestingly, we found that the treatment with CDDP and DOX also increased the nuclear fraction of c‐Abl (Figure 5C), in a similar manner induced by P5091, a specific inhibitor of USP7 (Figure 5D). These findings were consistent with c‐Abl‐induced apoptosis when it shuttles into nuclei.

It is reported that the Thr735 residue (T735) is critical for c‐Abl cytosolic retention,^25^ and we found when T735 was mutated, USP7 failed to increase cytosolic c‐Abl (Figure 5E). In contrast, USP7 induced c‐Abl‐G340L cytosolic retention (Figure 5F), further suggesting T735 is essential for c‐Abl cytosolic accumulation induced by USP7. Moreover, the 14‐3‐3α/β proteins are pivotal regulators of intracellular c‐Abl localization through binding with c‐Abl at the T735 residue.^25^ To find out whether USP7 promotes c‐Abl retention was due to its effects on the interaction between c‐Abl and 14‐3‐3α/β or the stability of 14‐3‐3α/β, we examined the role of the T735 residue in the binding of c‐Abl and the 14‐3‐3α/β, and the result demonstrated that the T735 residue was indispensable for their interaction because T735A markedly decreased the binding of 14‐3‐3α/β to c‐Abl (Figure 5G). Furthermore, when USP7 was overexpressed, 14‐3‐3α/β was upregulated and its binding to c‐Abl was significantly increased (Figure 5H,I). Conversely, knockdown of USP7 or treatment with P5091 markedly decreased 14‐3‐3α/β at the protein level in NSCLC cells (Figure 5J). Moreover, the IF assay also revealed that USP7 increased c‐Abl cytosolic retention (Figure 5K,L). All these results suggested that USP7 promotes cytoplasmic accumulation of c‐Abl in NSCLC cells by increasing its binding with 14‐3‐3α/β in a T735‐dependent manner.

### 3.6 USP7 activates c‐Abl and downstream signalling

**FIGURE 6:**
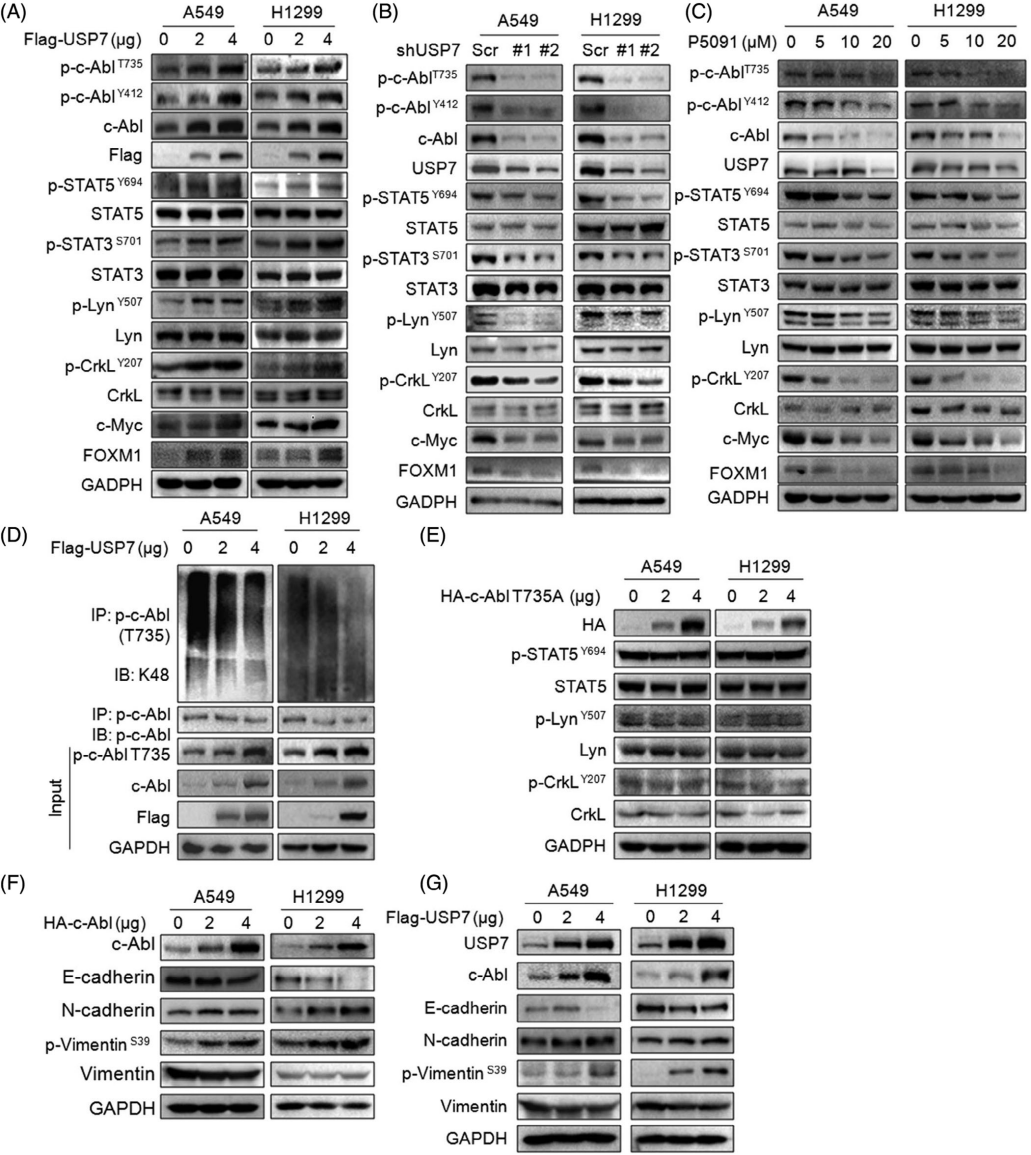
Thr735 is required for c‐Abl to be activated by USP7. (A‐B) NSCLC cells A549 and H1299 were transfected with USP7 plasmids (A) or infected with shUSP7 lentivirus (B) for 48 h, followed by IB assays as indicated. (C) A549 and H1299 cells were treated with P5091 for 24 h, followed by IB assay. (D) A549 and H1299 cells were transfected with USP7 plasmids for 48 h, followed by IP/IB assays as indicated. (E) A549 and H1299 were transfected with c‐Abl‐T735A plasmids for 48 h, followed by IB assay as indicated. (F and G) A549 and H1299 cells were transfected with c‐Abl (F) or USP7 (G) plasmids for 48 h, cell lysates were then subjected to IB assays as indicated.

As a non‐receptor tyrosine kinase, c‐Abl promotes tumour tumorigenesis and progression by activating a series of signal cascades including PLK1, CrkL and STAT.^7^, ^26^ Considering that USP7 stabilizes c‐Abl and increases its cytoplasmic retention, we subsequently examined the effects of USP7 on c‐Abl downstream signalling transduction. As shown in Figure 6A, ectopic expression of USP7 activated a series of c‐Abl‐related proteins, typically STAT3, STAT5, CrkL and Lyn in addition to c‐Abl itself. Furthermore, c‐Myc and FOXM1, two typical genes regulated by the c‐Abl signalling, were also upregulated (Figure 6A). In contrast, knockdown of USP7 or treatment with P5091 inactivated these proteins in NSCLC cells (Figure 6B,C). Interestingly, USP7 also activated c‐Abl phosphorylation at T735, and inhibition of USP7 by genetic or chemical manners suppresses T735 phosphorylation (Figure 6A–C). To explore the underlying mechanism, USP7 was transfected into NSCLC cells followed by the ubiquitination assay. The result indicated that USP7 decreased the polyubiquitination level of phospho‐c‐Abl (pT735) (Figure 6D), suggesting USP7 might also directly stabilize phosphorylated c‐Abl by preventing its quenching via the ubiquitin–proteasome pathway. Moreover, c‐Abl with T735 mutation failed to activate its downstream signalling proteins (Figure 6E). Moreover, previous studies have reported that activated ABL kinase can promote invadopodia formation in cancer cells, which further leads to EMT and activation of the subsequent metastatic cascade.^8^ We confirmed this finding in NSCLC cells by overexpressing c‐Abl (Figure 6F). We also found that USP7 activated these signalling molecules involved in cancer cell migration (Figure 6G). Taken together, these results demonstrated that USP7 activates c‐Abl signalling transduction by increasing its distribution in cytosol.

### 3.7 c‐Abl promotes NSCLC glycolysis by stabilizing HK2

**FIGURE 7:**
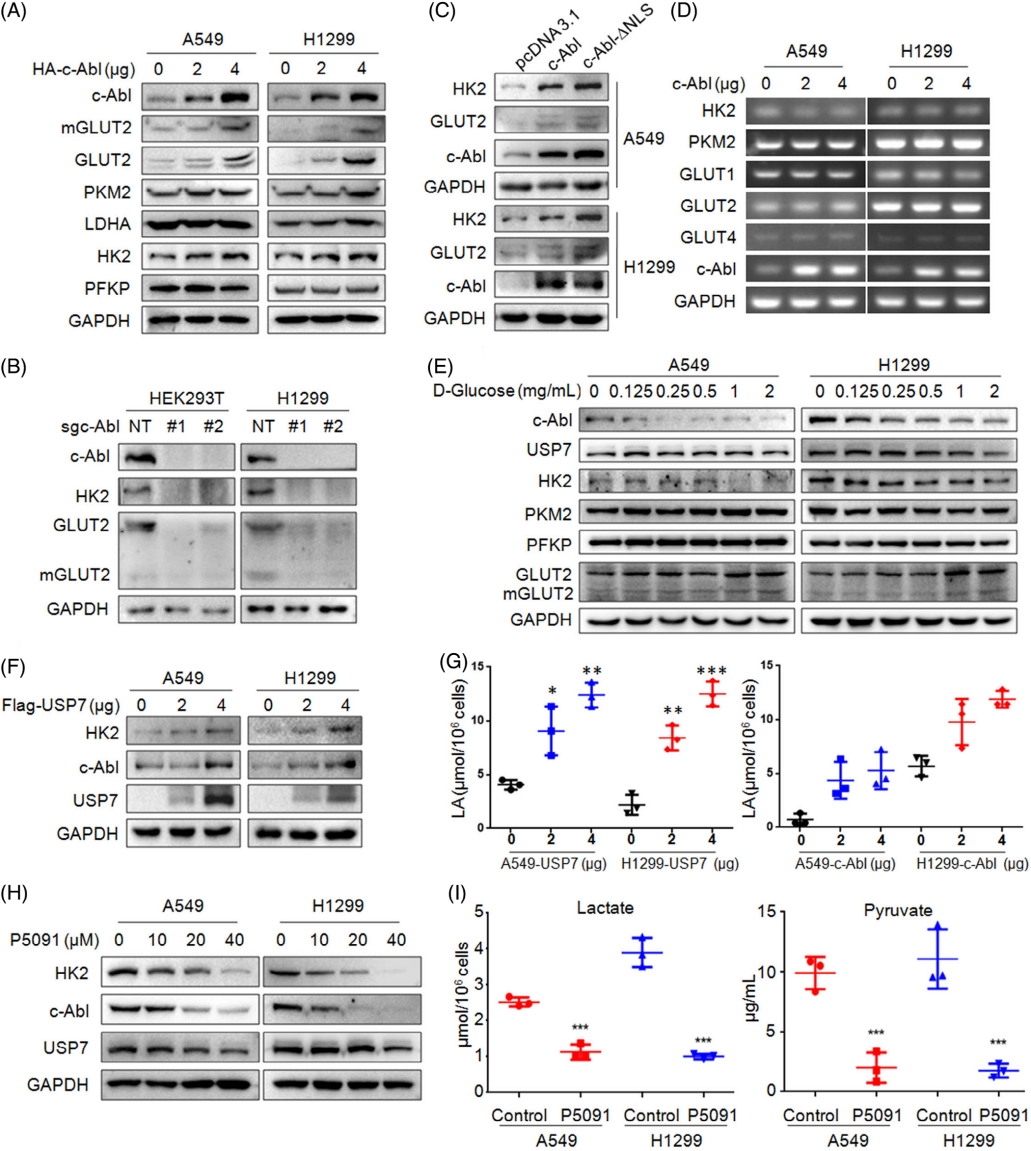
c‐Abl stabilizes HK2 and promotes NSCLC cell glycolysis. (A) A549 and H1299 were transfected with c‐Abl plasmids for 48 h, followed by IB assays against specific proteins as indicated. (B) c‐Abl was knocked out from HEK293T and H1299 cells, followed by IB assay. (C) All three NLSs were deleted from c‐Abl (c‐Abl‐∆NLS). This mutant (using its wild‐type c‐Abl as control) was transfected into A549 and H1299 cells for 48 h, followed by IB assays as indicated. (D) A549 and H1299 cells were transfected with c‐Abl plasmids for 48 h, followed by RT‐PCR assay. (E) NSCLC cells were cultured in medium without glucose for 24 h, and then glucose was added at different concentrations as indicated, cells were cultured for another 24 h, followed by IB assay. (F) A549 and H1299 cells were transfected with USP7 plasmids for 48 h, followed by IB assay. (G) A549 and H1299 cells were transfected with USP7 or c‐Abl plasmids for 48 h, followed by the measurement of lactate (LA). (H and I) A549 and H1299 cells were treated with P5091 for 48 h, followed by IB assay (H) and the measurement of lactate and pyruvate (I).

Cancer cells consume large amounts of glucose to quickly produce ATP through the ‘Warburg effect’ regardless of the presence of oxygen.^27^ In this process, the glycolysis rate is restricted by hexokinases, phosphofructokinase‐1 and pyruvate kinases.^28^ Given nuclear c‐Abl is able to induce glycolysis‐dependent podocyte apoptosis via interaction with transcription factor p53,^29^ we wondered whether USP7‐activated c‐Abl contributed to Warburg effect in NSCLC cells. To this end, cells were transfected with c‐Abl plasmids. The following assays showed that c‐Abl strikingly increased the protein levels hexokinase‐2 (HK2) and glucose transporter 2 (GLUT2), suggesting c‐Abl may promote cellular glycolysis (Figure 7A). Conversely, when c‐Abl was depleted by its sgRNAs, HK2 and GLUT2 were significantly decreased (Figure 7B). Moreover, this effect was independent of the NLS domain (Figure 7C), suggesting c‐Abl increased HK2 and GLUT2 proteins in cytoplasm, which was consistent with the oncogenic activity of c‐Abl. In addition, although c‐Abl possesses a DNA‐binding domain, it failed to upregulate the mRNA levels of both GLUT2 and HK2 (Figure 7D), indicating c‐Abl may increase their proteins by the post‐translational modification pathways. To our surprise, increased glucose downregulated c‐Abl and HK2, but not GLUT2 (Figure 7E), suggesting HK2 was more important in glycolysis modulated by c‐Abl. Furthermore, USP7 also increased HK2 in association with c‐Abl (Figure 7F). Both USP7 and c‐Abl increased the concentration of lactate, a typical product of glycolysis (Figure 7G). In contrast, P5091 decreased HK2 expression and inhibited the production of lactate and pyruvate in NSCLC cells (Figure 7H,I). All the results showed that the USP7/c‐Abl axis promotes NSCLC cell glycolysis by upregulating HK2.

### 3.8 c‐Abl upregulates HK2 by preventing its polyubiquitination while independent of AKT

**FIGURE 8:**
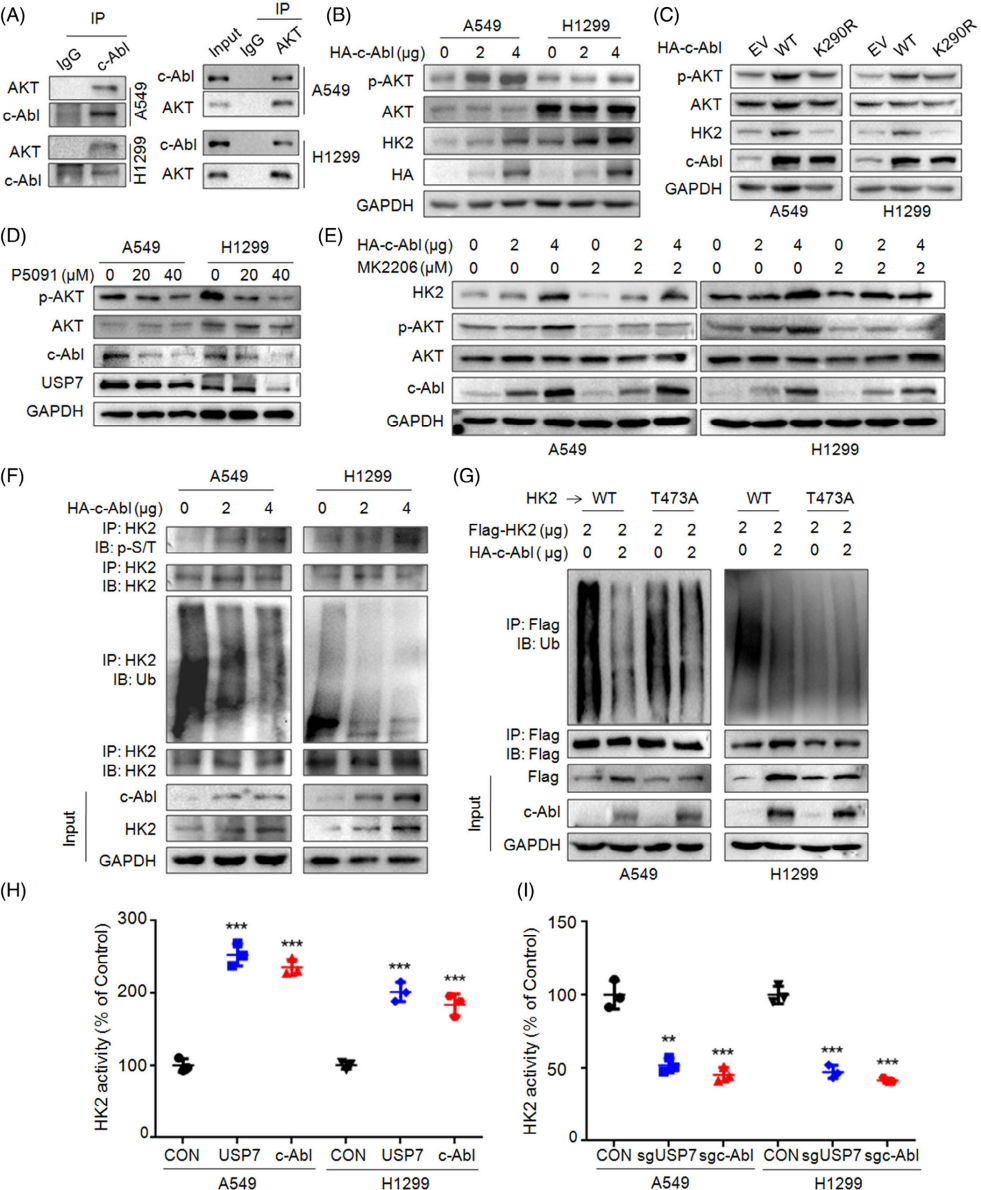
Thr473 is essential for c‐Abl to stabilize HK2 by preventing its polyubiquitination. (A) Cell lysates from NSCLC cell lines A549 and H1299 were subjected to reciprocal IP/IB assays as indicated. (B) A549 and H1299 cells were transfected with c‐Abl plasmids for 48 h, followed by IB assays. (C) A549 and H1299 cells were transfected with wild‐type (WT) c‐Abl or c‐Abl‐K290R plasmids or empty vector (EV) for 48 h, followed by IB assay. (D) A549 and H1299 cells were treated with P5091 for 48 h, followed by IB assay. (E) A549 and H1299 cells were transfected with c‐Abl plasmids for 24 h, followed by MK2206 treatment for 24 h, and then the cells were collected for IB assay. (F) A549 and H1299 cells were transfected with c‐Abl plasmids for 48 h, followed by IP/IB assays. (G) A549 and H1299 cells were transfected with HK2, HK2‐T473A or c‐Abl plasmids for 48 h, followed by IP/IB assays as indicated. (H and I) A549 and H1299 cells were transfected with c‐Abl or USP7 plasmids (H) or knocked down c‐Abl or USP7 (I) for 48 h, followed by measurement of HK2 activity. CON, control.

It has been demonstrated that AKT phosphorylates HK2 at T473, therefore upregulating its stability, inducing its mitochondrial localization and promoting tumorigenesis.^30^ Given c‐Abl is a kinase, we wondered whether it can phosphorylate AKT and then stabilize HK2. Therefore, we next checked the interaction between c‐Abl and AKT and the co‐IP assays turned out that c‐Abl indeed was bound to AKT (Figure 8A). Moreover, c‐Abl activated AKT in NSCLC cells (Figure 8B), but its inactive form (K290R) failed to upregulate HK2 and the phosphorylation level of AKT (Figure 8C). Therefore, these results suggested that c‐Abl was probably a kinase of AKT. Furthermore, P5091 downregulated the phosphorylation levels of AKT, HK2 and c‐Abl (Figure 8D), which was consistent with previous findings. However, in the presence of MK2206, an inhibitor of phosphorylated AKT,^31^ c‐Abl maintained its activity to upregulate HK2 (Figure 8E), suggesting that c‐Abl might act on HK2 independent of AKT. Furthermore, we found that c‐Abl increased HK2 phosphorylation, but decreased its polyubiquitination (Figure 8F). However, when T473 was mutated, HK2 ubiquitination was not modulated by c‐Abl (Figure 8G), suggesting that c‐Abl may also stabilize phosphorylated HK2. Lastly, both USP7 and c‐Abl promoted HK2 activity. When USP7 and c‐Abl were overexpressed, HK2 activity was strikingly increased (Figure 8H), while when either of the genes was knocked down, HK2 activity was markedly inhibited (Figure 8I). All the results indicated that c‐Abl upregulates HK2 stability and activity by phosphorylating and deubiquitinating HK2 independent of AKT.

### 3.9 USP7 and c‐Abl promotes NSCLC cell proliferation, migration and tumour growth in vivo

Our previous studies have revealed that USP7 acts as a Dub for c‐Abl ubiquitination, not only stabilizes c‐Abl, but also increases its presence in cytoplasm and activates its oncogenic signalling. To verify the pro‐cancer activity of USP7 in NSCLC, we evaluated USP7 in NSCLC growth, metastasis and invasion. The Transwell assays showed that overexpression of USP7 promotes NSCLC cell migration in both cell lines (Figure S1A), which was consistent with the previous finding that c‐Abl contributes to cancer metastasis.^8^ Subsequent EdU incorporation assays showed that USP7 and c‐Abl promoted NSCLC cell proliferation, while knockdown of these two genes inhibited cell proliferation (Figure S1B). Moreover, knockdown of USP7 and c‐Abl also inhibited NSCLC cell migration (Figure S1C). Collectively, these results indicated that USP7 and c‐Abl promote NSCLC cell migration and proliferation.

**FIGURE 9:**
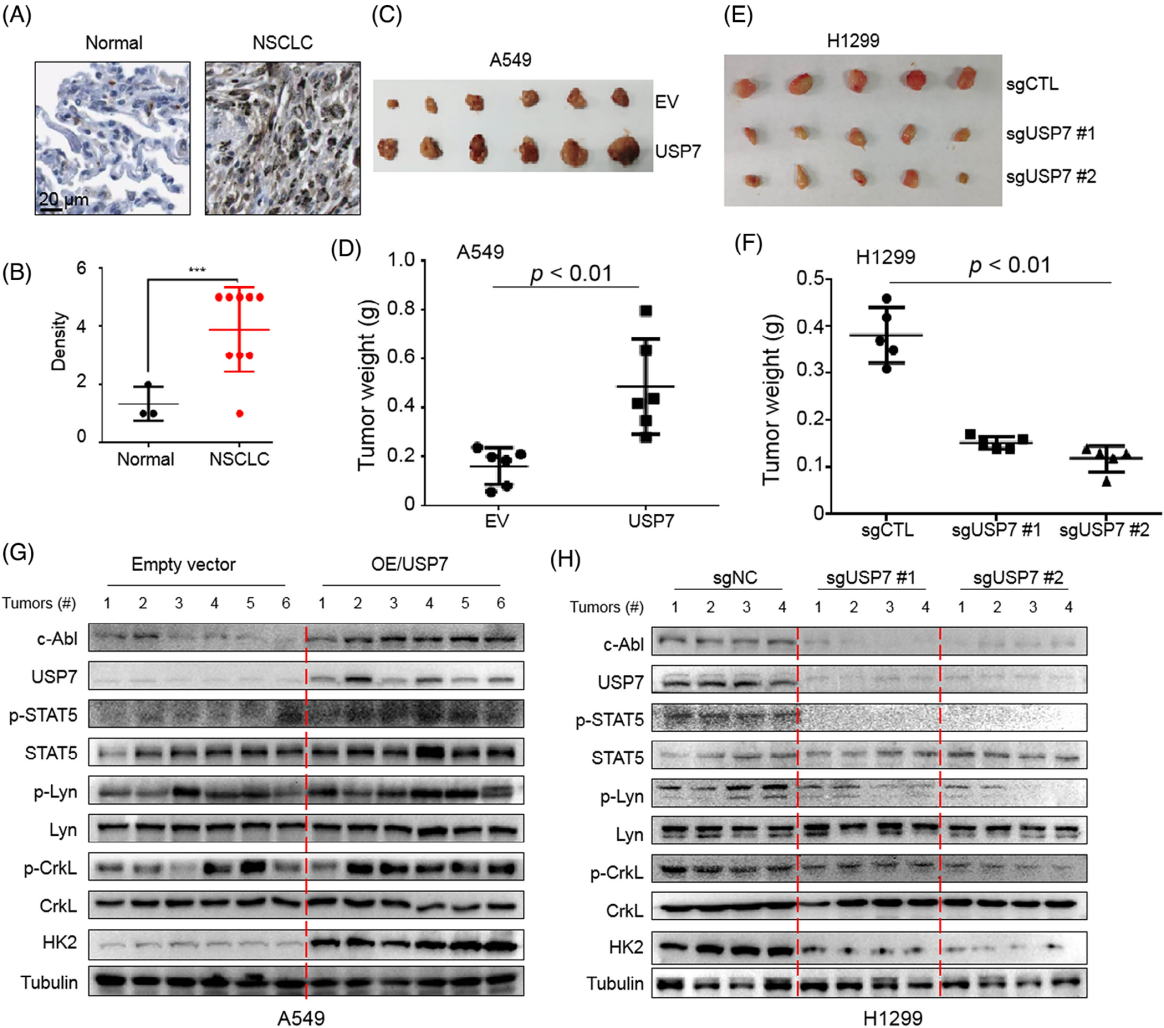
USP7 facilitates NSCLC xenograft tumour growth. (A and B) IHC analysis of the expression level of USP7 in normal lung tissues and tumours cited from the Human Protein Atlas. Representative images are shown. ***p < .001, Student's *t*‐test. (C and D) A549 stably expressing USP7 or the parental cells were subcutaneously injected in the right flanks of nude mice (*n* = 6), followed by monitoring tumour growth. (E and F) USP7 was knocked out from H1299 cells, and these cells were then injected into nude mice (*n* = 5). Tumour growth was monitored every other day. (G and H) Tumour tissues from treated mice were subjected to IB for indicated proteins.

To find out the activity of USP7 in NSCLC, we also evaluated the expression of USP7 in NSCLC tissues. As shown in Figure 9A,B, USP7 was highly expressed in NSCLC tissues. To further evaluate the effects of USP7 on NSCLC tumour growth in vivo, USP7‐deficient H1299 and USP7‐overexpressing A549 cells were injected subcutaneously into nude mice to establish xenograft models. The results showed that overexpression of USP7 promoted NSCLC tumour growth (Figure 9C,D). In contrast, when USP7 was knocked out, NSCLC tumour growth was strikingly decreased (Figure 9E,F). Tumour growth was highly consistent with the alterations of USP7, c‐Abl and its downstream signals including STAT5, Lyn, CrkL and HK2 in tumour tissues as shown in Figure 9G,H. The expression levels of USP7, c‐Abl and its downstream signals in xenografts were further examined with IHC staining assays, and the results were consistent with the IB findings (Figure S2A,B). All these results therefore demonstrated that USP7 activates c‐Abl, thus promoting NSCLC progression.

## 4 DISCUSSION

**FIGURE 10:**
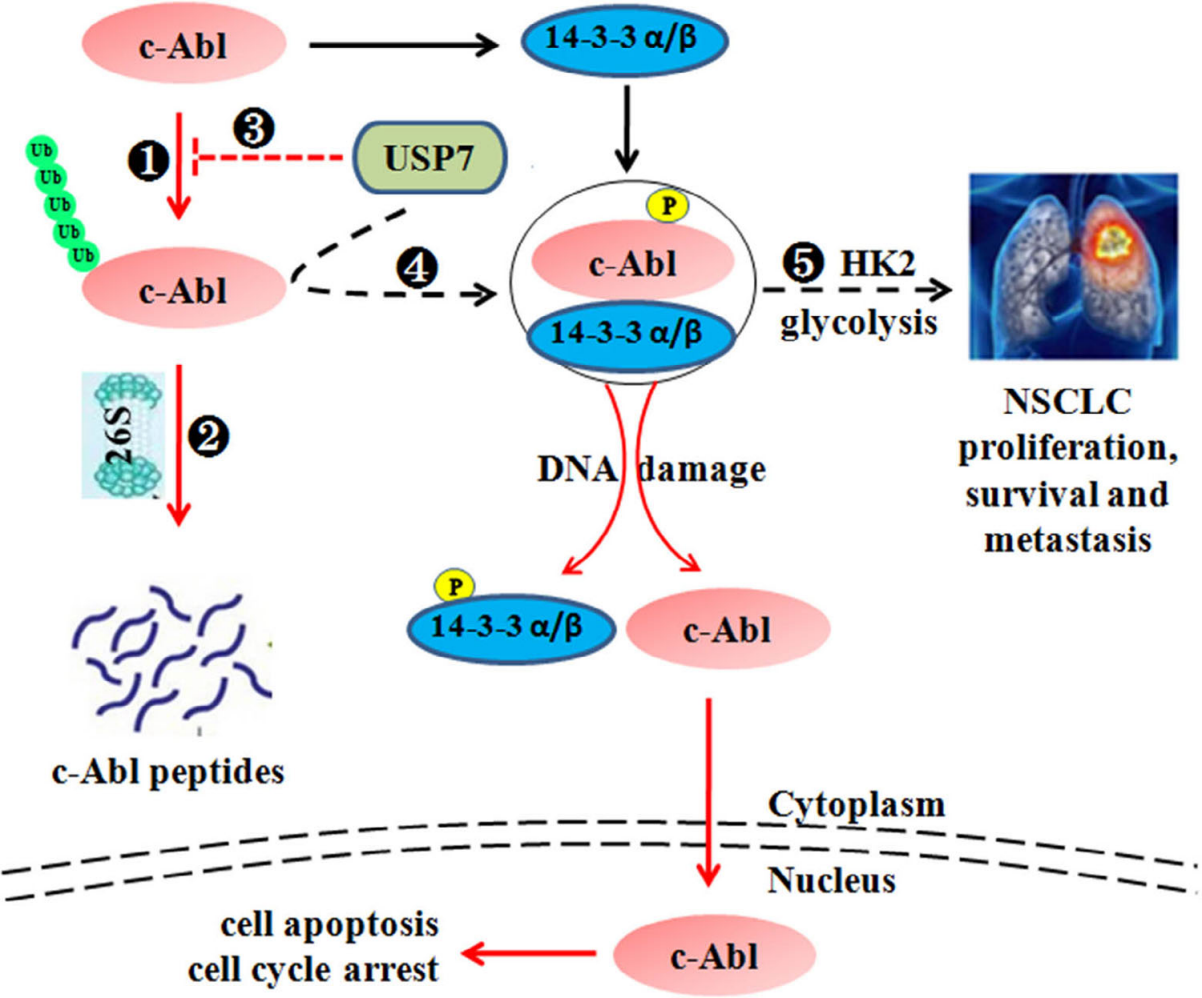
c‐Abl activity is modulated by USP7. c‐Abl is degraded via UPP (❶ and ❷). Overexpressed USP7 decreases the polyubiquitination of c‐Abl and promotes its stabilization (❸). Moreover, USP7 increases the binding of c‐Abl and 14‐3‐3α/β, inducing c‐Abl retention in cytosol (❹). c‐Abl also phosphorylates and stabilizes HK2 protein (❺), thereby facilitating cell glycolysis and promotes NSCLC survival and growth.

Our present study found that USP7 is a putative deubiquitinase of c‐Abl by hydrolyzing its K48‐linked polyubiquitination. USP7 also promotes the interaction between c‐Abl and 14‐3‐3α/β, thereby inducing c‐Abl accumulation in cytosol. Moreover, cytoplasmic c‐Abl phosphorylates and stabilizes HK2, inducing NSCLC cell glycolysis, and promotes NSCLC cell proliferation, survival and metastasis (Figure 10).

c‐Abl, a member of non‐receptor tyrosine kinases, plays a critical role in several physiological processes including cell apoptosis, cytoskeletal rearrangement, cell proliferation, cell transformation, cell cycle progression, DNA damage repair and tumorigenesis.^9^, ^32^ c‐Abl activity is also modulated by post‐translational modifications, including acetylation^33^ and ubiquitination.^10^ Ubiquitination is a major manner to modify proteins. Its known Smurf1 could act as a ubiquitin ligase for c‐Abl ubiquitination and proteasomal degradation.^10^ Simultaneously, protein ubiquitination is a dynamic process, and the linked ubiquitin chain could be removed by a specific deubiquitinase; however, the specific Dub of c‐Abl is not known. In the present study, by utilizing combined techniques and strategies, including the deubiquitinase library screen, the affinity‐purification coupled tandem MS and various biochemical assays, we identified that USP7 is a putative deubiquitinase of c‐Abl, which helps to understand the underlying mechanism in c‐Abl modulation by ubiquitination. USP7, or HAUSP, has been shown to modulate a variety of substrates, including both oncoproteins (such as RNF6)^12^ and tumour suppressor proteins (such as PTEN).^13^ However, different from its action on other substrates, USP7 stabilizes c‐Abl at both unphosphorylated and phosphorylated forms. Moreover, it not only stabilizes c‐Abl, but also arrests c‐Abl in cytosol, therefore promoting its oncogenic activity.

Previous studies have demonstrated that c‐Abl has three NLS domains and one NES domain, which leads to c‐Abl shuttle between nuclei and cytoplasm, therefore displaying different functions.^34^ Nuclear c‐Abl displays pro‐apoptotic activity upon DNA damage,^35^ while cytoplasmic c‐Abl is oncogenic by activating a series of oncoprotein substrates including STAT3 signalling.^7^, ^24^, ^36^ Although it is known that the chaperone protein 14‐3‐3α/β binds to c‐Abl and arrests it in cytosol, but the detailed mechanism is not well known. In the present study, we found the underlying mechanism is probably also associated with the stabilization and the binding of 14‐3‐3α/β to c‐Abl induced by USP7. This explanation is supported by the previous finding that shows, in the oncogenic context, 14‐3‐3α/β binds to c‐Abl and prevents it from nuclear import.^25^ In addition to stabilizing c‐Abl, USP7 also stabilizes 14‐3‐3α/β and phosphorylated c‐Abl. And we also revealed that T735 phosphorylation is essential for its binding to 14‐3‐3α/β and its cytosolic retention. Therefore, these findings reveal a novel mechanism underlying c‐Abl distribution in cytosol.

Tumorigenesis is highly related to the metabolic reprogramming in cancer cells.^37^ Tumour cells are predisposed to produce ATP through glycolysis at both hypoxia and normoxia to promote tumour cell growth.^38^ Hexokinases are the rate‐limiting enzymes in promoting glycolysism, and it has been upregulated in cancers by many factors such as activated ERBB2, RAS and AKT, as well as loss of p53.^39^ HK2, a key member of the HK family, catalyzes the first committed step in glucose metabolism by phosphorylating glucose, and it is specifically expressed in many foetal tissues and cancer cells. A recent study revealed that HK2 is aberrantly expressed in hepatocellular cancer cells (HCC) and promotes HCC tumorigenesis.^40^ In NSCLC cells, HK2 was found to promote glycolysis and chemoresistance of lung cancer cells.^41^ Consistent with these findings, in our study, theUSP7/c‐Abl axis promotes NSCLC cell glycolysis by increasing the lactate and pyruvate production by upregulating HK2 protein stability and phosphorylation. Although HK2 interacts with AKT, a known kinase and stabilizer of HK2,^30^ HK2 can also be upregulated by c‐Abl independent of AKT, because c‐Abl still increases HK2 in the presence of MK2206, a specific inhibitor of AKT. These findings suggest that c‐Abl might be a direct kinase of HK2. Notably, we found that c‐Abl can also downregulate the polyubiquitination level of HK2 in a manner dependent of its T473, because T473A of HK2 failed to be well ubiquitinated. Therefore, we found that c‐Abl is a kinase of HK2 and promotes NSCLC cell glycolysis.

In summary, the present study identifies USP7 is a putative Dub for c‐Abl K48‐linked polyubiquitination. Moreover, USP7 promotes the presence of c‐Abl in cytoplasm by increasing its binding with 14‐3‐3α/β protein. The USP7/c‐Abl axis stabilizes HK2 and promotes NSCLC cell glycolysis, inducing NSCLC proliferation and survival (Figure 10). Within our knowledge, this is the first report on the deubiquitinase of c‐Abl. This study also highlights a novel regulation in USP7/c‐Abl/HK2 signalling. Targeting the USP7/c‐Abl/HK2 axis may provide a new strategy for the treatment of NSCLC.

## AUTHOR CONTRIBUTIONS

X.M., Y.H. and Z.H. designed the study; Y.H., S.J., X.W., Y.C., Y.Z., J.L. and Y.S. conducted experiments. X.M. and Z.H. analyzed data; Z.Z provided key agents. X.M. and Y.H. wrote the manuscript.

## CONFLICT OF INTEREST STATEMENT

The authors declare that they have no conflicts of interest.

## ETHICAL APPROVAL

This animal experiment was approved by the Review Board for Animal Welfare and Ethics of Guangzhou Medical University. The study on primary NSCLCs was approved by the Ethical Committee of Guangzhou Medical University.

## Supporting information

Supporting Information

## Data Availability

The data that support the findings of this study are available from the corresponding author upon reasonable request.
