# Supplementary material for: USP7 promotes non‐small‐cell lung cancer cell glycolysis and survival by stabilizing and activating c‐Abl
Source: Clin Transl Med. 2023 Dec 11;13(12):e1509. doi: 10.1002/ctm2.1509 (PMC10713873; doi:10.1002/ctm2.1509)
Supplement: Supplementary file 1 — Supporting Information [file CTM2-13-e1509-s001.docx]

**USP7 promotes non-small cell lung cancer cell glycolysis and survival by stabilizing and activating c-Abl**

Yuanming He^1,2^, Shuoyi Jiang^1^, Xiaoge Wang^1^, Yaoli Cui^1^, Yueya Zhong^1^, Jingpei Liang^1^, Yuning Sun^1^, Zhigang Zhu^3^, Zhenqian Huang^1^, Xinliang Mao^1,2*^

^1^ The First Affiliated Hospital & Guangdong and Guangzhou Key Laboratory of Protein Modification and Degradation, School of Basic Medical Sciences, Guangzhou Medical University, Guangzhou 511436, P. R. China

^2^ GMU-GIBH Joint School of Life Sciences, The Guangdong-Hong Kong-Macau Joint Laboratory for Cell Fate Regulation and Diseases, Guangzhou Medical University, Guangzhou 511436, P. R. China

^3^ Division of Hematology & Oncology, Department of Geriatrics, Guangzhou First People's Hospital, College of Medicine, South China University of Technology, Guangzhou, Guangdong 510180, P. R. China

 **Supplemental Fig. 1. USP7 and c-Abl promote NSCLC cell proliferation and migration.** (**A**) NSCLC cells A549 and H1299 were transfected with USP7 plasmids for 48 hrs, followed by Transwell assay. (**B**) A549 and H1299 cells were transfected with c-Abl or USP7 plasmids or their siRNA for 48 hrs, followed by EdU incorporation assay and statistical analysis. (**C**) A549 cells were transfected with c-Abl or USP7 siRNA for 48 hrs, followed by Transwell assay.

 **Supplemental Fig. 2. IHC analysis of indicated proteins in NSCLC xenografts in nude mice.** A, Tumors derived from USP7-overexpressing A549 cells were subjected to IHC assays against indicated proteins. B, Tumors derived from USP7-knockingout H1299 cells were subjected to IHC assays against indicated proteins.
